# Higher-order regression three-dimensional motion-compensation method for real-time optical coherence tomography volumetric imaging of the cornea

**DOI:** 10.1117/1.JBO.27.6.066006

**Published:** 2022-06-24

**Authors:** Ruizhi Zuo, Kristina Irsch, Jin U. Kang

**Affiliations:** a Johns Hopkins University, Whiting School of Engineering, Baltimore, Maryland, United States; b Vision Institute, CNRS, Paris, France; c Johns Hopkins University, School of Medicine, Baltimore, Maryland, United States

**Keywords:** optical coherence tomography, motion compensation, real-time, cornea, volumetric imaging

## Abstract

**Significance:**

Optical coherence tomography (OCT) allows high-resolution volumetric three-dimensional (3D) imaging of biological tissues *in vivo*. However, 3D-image acquisition can be time-consuming and often suffers from motion artifacts due to involuntary and physiological movements of the tissue, limiting the reproducibility of quantitative measurements.

**Aim:**

To achieve real-time 3D motion compensation for corneal tissue with high accuracy.

**Approach:**

We propose an OCT system for volumetric imaging of the cornea, capable of compensating both axial and lateral motion with micron-scale accuracy and millisecond-scale time consumption based on higher-order regression. Specifically, the system first scans three reference B-mode images along the C-axis before acquiring a standard C-mode image. The difference between the reference and volumetric images is compared using a surface-detection algorithm and higher-order polynomials to deduce 3D motion and remove motion-related artifacts.

**Results:**

System parameters are optimized, and performance is evaluated using both phantom and corneal (*ex vivo*) samples. An overall motion-artifact error of <4.61 microns and processing time of about 3.40 ms for each B-scan was achieved.

**Conclusions:**

Higher-order regression achieved effective and real-time compensation of 3D motion artifacts during corneal imaging. The approach can be expanded to 3D imaging of other ocular tissues. Implementing such motion-compensation strategies has the potential to improve the reliability of objective and quantitative information that can be extracted from volumetric OCT measurements.

## 1 Introduction

First introduced 30 years ago, optical coherence tomography (OCT)^1^ has become a powerful medical imaging modality with micrometer resolution. It allows high-speed volumetric three-dimensional (3D) imaging of biological tissues and has also been gaining a lot of interest as an intraoperative imaging modality to guide microsurgeries.^2^^–^^4^ However, because a typical 3D-image acquisition speed is in the order of a few hertz, it often suffers from motion artifacts due to involuntary and physiological movements of living tissue.^5^ In particular, in ophthalmology, where OCT found its first uses and remains the primary field of application,^6^ the target is constantly in motion.^7^ This is because, even during so-called steady fixation on a target, there are involuntary fixational eye movements (e.g., tremors, drifts, and microsaccades).^8^ Aside from such involuntary lateral ocular motion, another major source of involuntary eye movement is from vascular pulsation or respiration that manifests itself as motion in the axial direction^9^ and may have a much more direct effect on the overall distortion of OCT images.^5^ Any such involuntary eye movements may also be exaggerated in patients^10^ and limit the reproducibility of quantitative measurements.

While a wealth of methods has been developed and explored to compensate for ocular motion during OCT imaging of the retina (both hardware-based^11^^,^^12^ and software-based solutions^13^^–^^18^), fewer works have focused on motion-compensated OCT-based measurements of the cornea.^5^^,^^19^^,^^20^ Most of those are targeted toward corneal topography applications,^19^^,^^20^ assume the surface to be perfectly spherical^20^ or use a spherical model to estimate corneal curvature,^5^ and are based on time-costly postprocessing algorithms.^5^^,^^18^^,^^19^

In this paper, we propose and analyze a motion-compensated 3D-OCT approach geared toward real-time volumetric corneal imaging including in patients. Our approach is based on using a combination of intensity and topological analysis, where the tissue surface is identified and fitted to a polynomial curve and compared with three orthogonal references initially obtained to deduce the magnitude of motion-related image shift. The motion artifact along each (fast-axis) B-mode image is deduced by fitting these shift distances from the reference (slow-axis) images using high-order polynomials. This result is then used to compensate for the motion artifacts during the entire volumetric imaging procedure. To test and optimize system performance, we applied our algorithm to both corneal phantom and *ex vivo* bovine samples.

## 2 Method

### 2.1 3D-OCT System

**Fig. 1:**
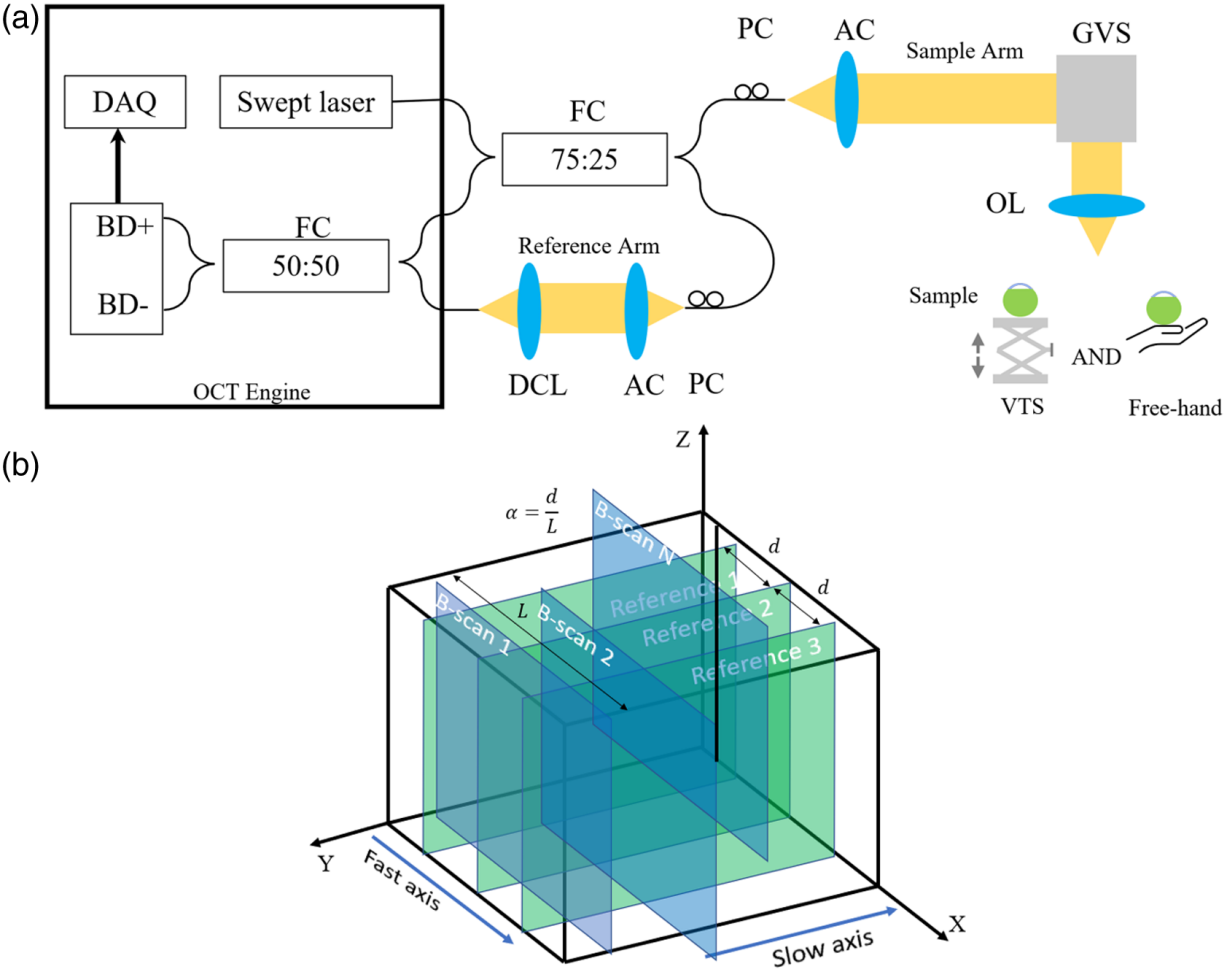
(a) A schematic representation of the OCT system setup. FC, fiber coupler; PC, optic fiber polarization controller; AC, achromatic collimator; GVS, galvanometer scanners that contain two scanning mirrors; OL, objective lens; VTS, vertical translation stage; DCL, dispersion compensation lens; BD+, BD-, balanced detector; DAQ, data acquisition board. (b) Schematic of the scanning protocol of the motion-compensated 3D-OCT system. d is the distance between adjacent reference images, L is the width of the B-scan image, and α is defined as the ratio between d and L.

The 3D motion-compensated OCT system is based on an in-house built 100 kHz swept-source OCT (Axsun) system operating at the center wavelength of λ=1060  nm and a 110 nm tuning range. Figures 1(a) and 1(b) illustrate the OCT setup and proposed scanning protocol of the motion-compensated 3D-OCT system, respectively. The output of the swept laser is split into two arms by a 75:25 fiber coupler (TW1064R3A2A, Thorlabs). About 25% of the light is coupled to the sample arm and the other 75% is coupled to the reference arm. Polarization controllers (FPC020, Thorlabs) are used to adjust the polarization states of the two arms, enabling control of the interference between them. Achromatic collimators (AC300, Thorlabs) are utilized to collimate the light from the fiber. The collimated light is focused on the sample using an objective lens (LSM04, Thorlabs) with a numerical aperture (NA) of 0.05, corresponding to the lateral resolution of the OCT system of 0.61λ/NA=12.9  μm. A vertical translation stage (VTS) (19089, Cenco–Lerner) allows simulation of axial sample motion (maximum around 1.5 cm) by precisely translating the stage using a micrometer. To simulate involuntary and random sample motion occurring in both lateral and axial dimensions, the sample was positioned in the palm of a hand. Galvanometer scanners (GVS002, Thorlabs), which contain two scanning mirrors are used to scan along the so-called “fast-axis” (X) and “slow-axis” (Y) to generate the volumetric data. The C-scan data composed of 1024×512×1024  voxels (X×Y×Z) corresponds to a 1  cm×1.1  cm×3.7  mm volume in corneal tissue. Note that the axial resolution, defined by the spectral bandwidth of the system, was experimentally confirmed to be 6  μm in air and 4.5  μm in corneal tissue. A dispersion compensation lens (LSM04DC, Thorlabs) is employed to compensate for the dispersion induced by the objective lens. Reference- and sample-arm signals are mixed by a 50:50 coupler (TW1064R3A2A, Thorlabs) and detected by a balanced detector. With future *in vivo* applications in mind, the power of the probe beam at the output (on the sample) was intentionally reduced to around 1 mW, to ensure light levels during imaging well below the maximum permissible exposure for ocular tissues. The signal-to-noise-ratio at the imaging depth is >55  dB. The interferometric data is then processed by a data acquisition board (500 MSPS, 12-bit resolution) with a digitizer (embedded in the OCT engine from Axsun) before being transferred to a computer (Intel i7-9700F, Nvidia GeForce RTX 2060 SUPER, 16GB RAM) via a PCIe port by a frame grabber (PCIe-1433, National Instrument, Austin, Texas, United Statess) for further processing. Custom software written in C++/C# and a graphics processing unit (GPU) is used for high-speed data acquisition and processing,^3^^,^^21^^,^^22^ resulting in a real-time B-scan rate of 100 Hz.

The proposed motion-compensated 3D-OCT system scanning protocol, as shown in Fig. 1(b), first scans three B-scans in the slow axis to obtain reference images, which are composed of 512×1024  pixels (Y×Z). This is immediately followed by a standard C-scan, scanning along X and Y, to collect volumetric images of the moving sample. The d is defined as the distance between the initial reference scans along the slow axis. L is the width of the B-scan image and α is defined as the ratio between d and L.

### 2.2 Workflow and Algorithm

**Fig. 2:**
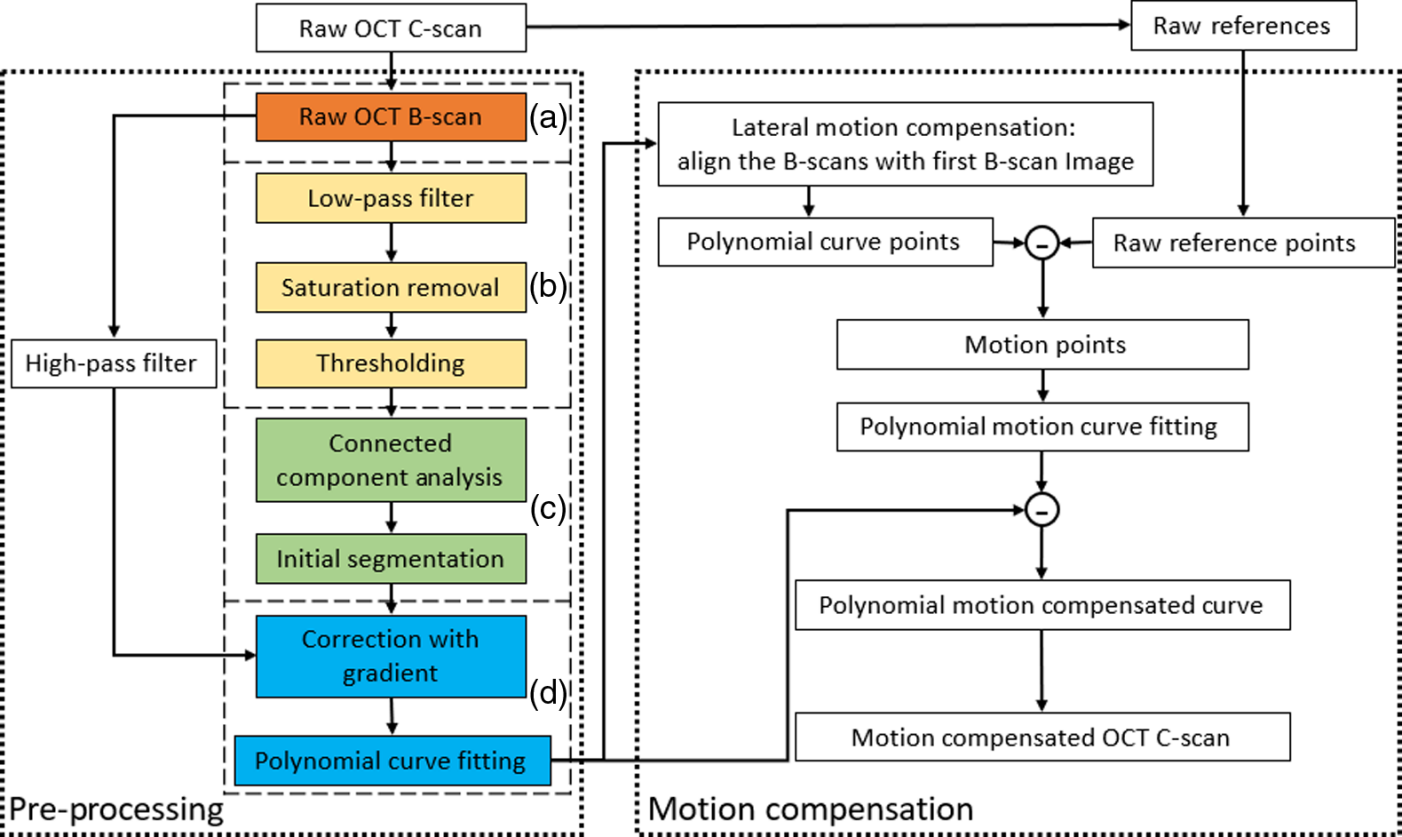
Flowchart of the motion-compensated OCT system algorithm, with (left) preprocessing and (right) motion-compensation steps. (a)–(d) The preprocessing procedure is employed to segment the sample’s surface boundary and fit to a higher-order polynomial. The motion-compensation procedure is based on this preprocessing result to deduce the sample motion by comparing it with the reference images, and then generates a motion-free C-scan by shifting every preprocessed B-scan accordingly.

After obtaining three B-scan reference images along the slow axis and as the C-scan data is being obtained, each B-scan image in the volumetric data is processed using a custom algorithm, first developed in MATLAB as a testbed prototype, and then programmed in C++ for real-time processing. The algorithm, as summarized in the flowchart depicted in Fig. 2, consists of two main threads, namely preprocessing and motion compensation, that are detailed in the following Secs. 2.2.1–2.2.3. Briefly, the preprocessing procedure is used to segment the surface boundary from the sample and fit to a higher-order polynomial curve. The motion-compensation procedure is based on this preprocessing result and uses it to deduce the sample motion by comparing it to the reference images. The effect of the motion is then subtracted from the image to display motion-free volumetric (C-scan) images. When multiple consecutive volumes are imaged, a new set of three references is acquired to achieve motion correction for each volume.

#### 2.2.1 Preprocessing algorithm

Before the thresholding can be applied, we first use a 10×10 Gaussian filter with a standard deviation of 4 for every B-scan image ($I_{0}$) to smooth the speckle pattern. Then, we remove the saturation artifacts introduced by light reflected from a highly specular surface that are over the dynamic range of the data-acquisition system. We simplified the saturation removal method based on the minimum variance mean-line subtraction described in Refs. 23 and 24, to reduce the computation cost. Specifically, I, the intensity value of the saturation-free image, is assigned as

$$I=I_{g}-I_{g}*A,$$ (1)

where $I_{g}$ is the Gaussian-filtered image and A is the spatially averaging filter (size 40×1) with a value of 1/40.

**Fig. 3:**
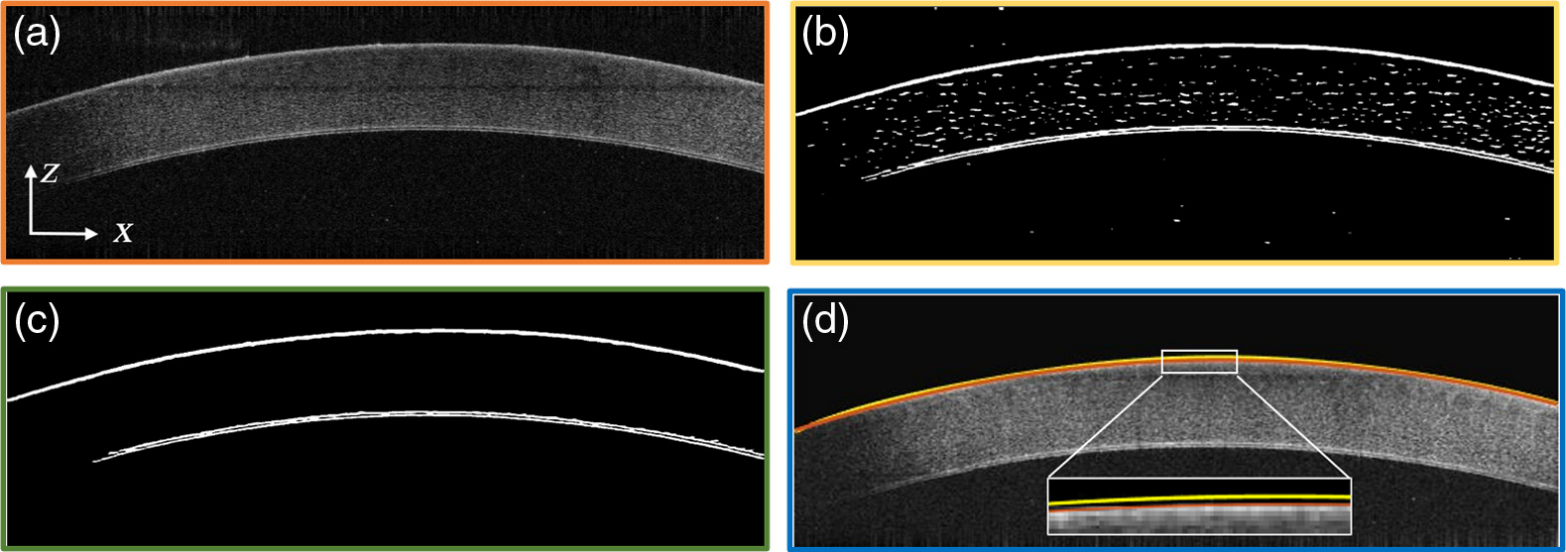
OCT images showing the result of the preprocessing steps, using a bovine corneal sample. (a) Original B-scan image before any preprocessing. Scale bar: 1 mm for z axis and 10 mm for x axis. B-scan image (b) after applying the thresholding step, (c) showing the result of the connected component analysis, and (d) showing the final segmentation result, with the red line indicating the polynomial fitting curve of the corneal surface. The yellow line indicates the initial segmentation result based on the connected component analysis for comparison purposes.

Next, to facilitate connected component analysis, we convert every grayscale B-scan into a binary image, based on a threshold value. More precisely, we applied the adaptive thresholding method^25^ to calculate the thresholding value, and this ensures that the sample surface constitutes the largest area of bright components in the image after thresholding. Figure 3(a) shows the *ex vivo* bovine B-scan image. Figure 3(b) shows the corneal B-scan image after this thresholding was applied and clearly shows the upper and lower boundaries of the cornea sample.

The subsequent connected component analysis selects the regions with the largest connected elements of the maximal intensity in the image and erases the remaining bright and isolated pixels, retaining the basic shape of the sample boundaries, as illustrated in Fig. 3(c). To further improve this initial segmentation result, which is obtained from low-pass (Gaussian) filtered images, we also high-pass filtered the original B-scan images to obtain tissue boundaries. This result is used to further refine the segmented boundary result. Specifically, we first obtain high-pass filtered images (F) by applying a combination of Sobel filter, $S={\left[1,2,1;0,0,0;-1,-2,-1\right]}^{T}$, and Prewitt filter, $P={\left[1,1,1;0,0,0;-1,-1,-1\right]}^{T}$ as follows:

$$F=\sqrt{{\left(S*I_{0}\right)}^{2}+{\left(P*I_{0}\right)}^{2}}.$$ (2)

Then, we search the high-pass filtered image, in the vicinity (±15  pixels) of the initially segmented surface boundary [yellow line, Fig. 3(d)], for the pixel with the highest gradient (both horizontally and vertically) and use the result to replace the initial segmentation result. The resultant, corrected sample surface segmentation is fitted with a higher-order polynomial curve [red line, Fig. 3(d)]. The polynomial fitting algorithm uses the pixel location to form a Vandermonde matrix (V), resulting in a linear system VP=Z, which is also

$$(\begin{array}{cccc} x_{1}^{n} & x_{1}^{n-1} & \cdots & 1\\ x_{2}^{n} & x_{2}^{n-1} & \cdots & 1\\ \vdots & \vdots & \ddots & \vdots \\ x_{m}^{n} & x_{m}^{n-1} & \cdots & 1 \end{array})(\begin{array}{c} p_{1}\\ p_{2}\\ \vdots \\ p_{n+1} \end{array})=(\begin{array}{c} z_{1}\\ z_{2}\\ \vdots \\ z_{m} \end{array}).$$ (3)

Note that we define the points on the boundary with $\left(x_{i},z_{i})(i=1,\;2,\ldots ,m\right)$, where m is the number of points and n is the order of the polynomial. The coefficients of the polynomial are solved by $P=(V^{T}V^{-1})V^{T}Z$, the matrix multiplication between the Moore–Penrose inverse of V and Z.

#### 2.2.2 Lateral motion-compensation algorithm

The lateral motion-compensation algorithm is based on the preprocessed (segmented) result. We define the first B-scan cornea surface ($z_{1}$) as the target surface, then move every other B-scan surface ($z_{t}$, t is the index of B-scan) horizontally to align it with the target surface. For each B-scan, the n’th order polynomial fitting curve of the corneal surface can be expressed as

$$z(x)=p_{n+1}x^{n}+p_{n}x^{n-1}+\cdots +p_{1}.$$ (4)

The lateral movement of t’th B-scan could be figured out by minimizing cost function

$$\Delta l_{t}=\underset{\Delta l_{t}}{\arg\;\min}(\mathrm{var}(|z_{t}(x_{1}+\Delta l_{t})-z_{1}(x_{1})|,|z_{t}(x_{2}+\Delta l_{t})-z_{1}(x_{2})|,\ldots ,|z_{t}(x_{m}+\Delta l_{t})-z_{1}(x_{m})|)),$$ (5)

in which, $\Delta l_{t}$ is the lateral movement, var represents variance, and m is the total number of A-scan (1024 in this case).

**Fig. 4:**
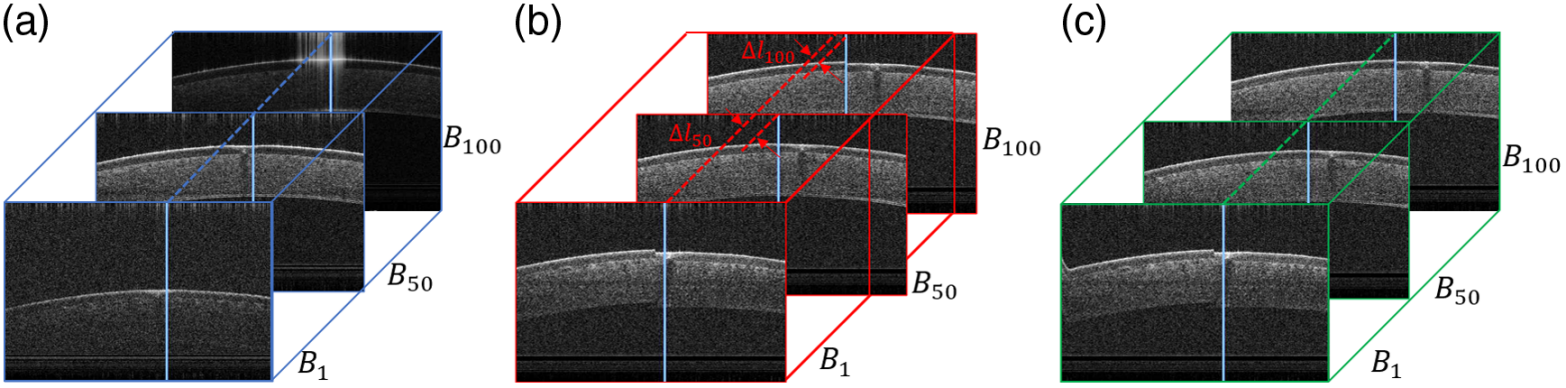
Pictorial explanation of lateral motion compensation by means of an *ex vivo* bovine cornea. (a) Three static B-scan images serving as a reference. (b) and (c) B-scan images pre- and post-lateral motion compensation, respectively.

Figure 4 illustrates the lateral motion-compensation mechanism. Figure 4(a) depicts three examples B-scan images (1st, 50th, and 100th) from a volumetric OCT data set of a static *ex vivo* bovine corneal sample. Figure 4(b) shows the same bovine corneal B-mode images with indicated lateral movement. Figure 4(c) shows the lateral motion-compensated result of (b). The solid blue line in every B-mode image indicates the location of the corresponding corneal surface peak. The dashed blue, red, and green lines in Figs. 4(a)–4(c) are used to illustrate the relative lateral shifts of different B-scans [e.g., $\Delta l_{50}$ and $\Delta l_{100}$ in Fig. 4(b)]. About 50 pixels of both lateral image borders are cropped to remove the empty A-scans generated by the compensation.

#### 2.2.3 Axial motion-compensation algorithm

**Fig. 5:**
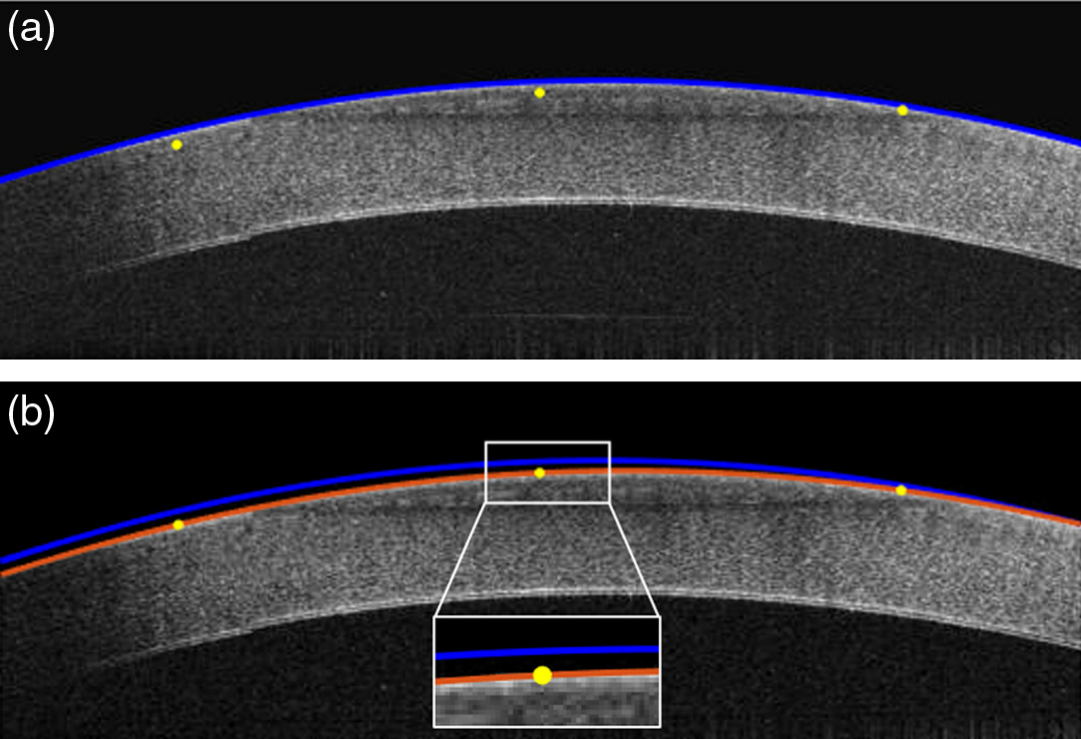
Axial motion-compensation procedure illustrated by means of an *ex vivo* bovine cornea. (a) Preprocessed and laterally-compensated B-scan image before axial motion compensation, showing the higher-order polynomial fit (blue line), z(x), obtained from preprocessing and the three reference points (yellow dots), $r_{i}(x_{i},y_{i},z_{i})(i=1,2,3)$, collected from the intersection planes. (b) B-scan image after axial motion compensation, with corresponding final surface fit (red line), f(x).

The axial motion-compensation algorithm uses the lateral motion-compensated result and is illustrated in Fig. 5. We first compare the B-scan surface detection result with the three reference images and obtain the differences in the axial position at the three intersecting planes of each B-scan. Then, the axial shift for every A-scan within the B-scan is estimated by fitting it with a polynomial and the shift is compensated accordingly in the final images. The motion-free volumetric image can be generated from sequentially-processed B-scans.

The three reference points [yellow dots in Fig. 5(a)] are defined as $r_{i}(x_{i},y_{i},z_{i})(i=1,2,3)$, which we write as $r_{i}(x_{i}^{r},z_{i}^{r})(i=1,2,3)$, assuming all points in the same B-scan images share the same y-axis coordinate. Then, we can deduce the axial motion at the three reference points, $a(x_{1}^{r})$, $a(x_{2}^{r})$, and $a(x_{3}^{r})$ as

$$a(x_{i})=z(x_{i}^{r})-z_{i}^{r},\;(i=1,2,3).$$ (6)

The axial motion at every (other) points of z(x), defined as m(x) is estimated by generating a second-order polynomial function based on the three reference points. Finally, we axially shift the location of every A-line in the B-scan to obtain the motion-compensated result [red line in Fig. 5(b)] f(x) as

f(x)=z(x)−a(x). (7)

## 3 Results

We optimized system parameters and evaluated system performance mainly using phantom (thin plastic shell) and corneal (*ex vivo* bovine) samples. In addition, the order of polynomial for the surface fit was optimized using clinical images from a commercial ophthalmic OCT system (Avanti, Optovue) to assure it will work with various surface topographies.

Tissue motion was simulated in two different ways. First, by positioning the samples on the vertical translation stage [VTS, Fig. 1(a)] and manually moving the stage up and down, axial motion was generated at frequencies ranging from about 0 to 5 Hz, with a typical amplitude around 1 mm. We know the frequency range of axial ocular motion could be from 0 to 125 Hz.^5^ However, the amplitude of motion decreases rapidly with higher frequency and the axial resolution of our OCT system is 4.5 microns so that any smaller motions occurring at frequencies above about 5 Hz are not detectable with our system. Second, samples were positioned in the palm of the hand; such “free-hand” motion served to simulate the involuntary 3D motion of living tissue occurring with random frequencies and amplitudes.

### 3.1 Optimizing System Parameters

#### 3.1.1 Determining order of polynomial for corneal surface fit

**Fig. 6:**
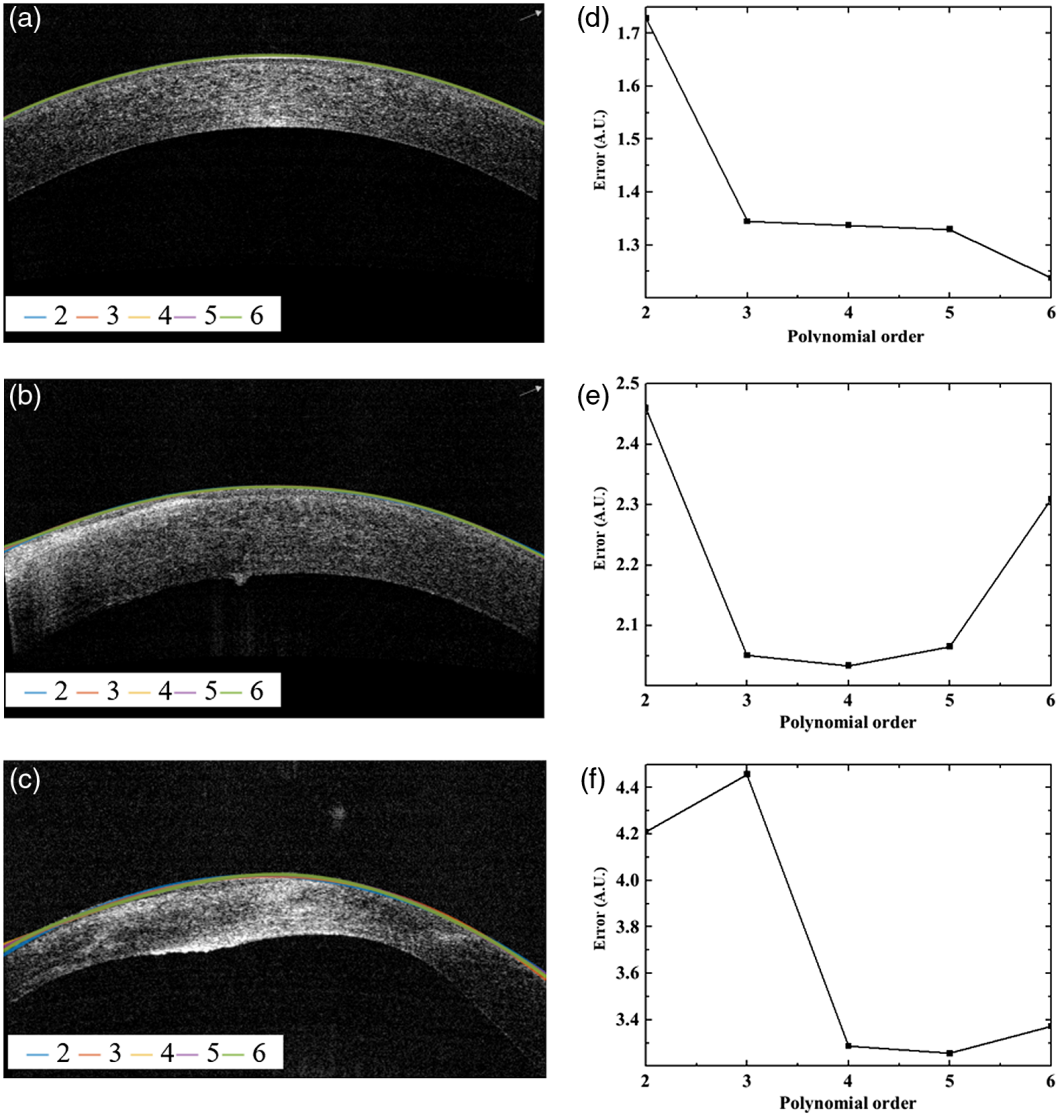
Determining the optimal order of polynomial for the segmented corneal surface fit during the preprocessing step in Fig. 2(d), using clinical SD-OCT (Avanti, Optovue) images of corneas with normal and abnormal surface topology. (a)–(c) The curve fitting with the different order of polynomials (from second to fourth order, as indicated by different colors); (d)–(f) corresponding fitting error, i.e., the RMSE between polynomial fit and manually segmented surface, of the (d) healthy cornea, (e) cornea with bullous keratopathy, and (f) cornea with keratoconus.

With the proposed OCT system being geared toward ophthalmic applications in patients (e.g., to guide ocular surgeries and/or to extract quantitative information from volumetric measurements), our algorithm must work not only on normal corneas but also on pathological ones with abnormal surface topography. We hence used representative clinical OCT images (Avanti, Optovue), including from patients with bullous keratopathy and keratoconus to determine the optimal order of polynomial for the final surface fit during preprocessing of the data. Images were collected retrospectively; however, all subjects provided informed consent to have their images used in research, which was approved by the Institutional Review Board (Patient Protection Committee, Ile-de-France V) and adhered to the tenets of the Declaration of Helsinki. We applied different polynomial fits ranging from second- to sixth-order to the data (image) sets, during step (d) of the preprocessing algorithm [Fig. 2(d)]. Figure 6 shows the results for three typical examples from each group, i.e., for a healthy cornea [Fig. 6(a)], a cornea with bullous keratopathy [Fig. 6(b)], and a cornea with keratoconus [Fig. 6(c)].

We used the fitting error, more precisely the root mean square error (RMSE) representing the difference between the surface fit and the ground truth, with the latter being estimated by manually segmenting the corneal surface, to quantify the results. The resulting fitting error for the three representative corneal images as a function of polynomial order is depicted in Figs. 6(d)–6(f). As expected, the fitting error decreases with increasing polynomial order for the healthy cornea with normal surface topography [Fig. 6(d)]. However, this is not the case for the diseased corneas with abnormal surface pathology [see Figs. 6(e) and 6(f)], where one can note the error starting to increase again for the fifth- and sixth-order polynomials for the cornea with bullous keratopathy [Fig. 6(e)] and keratoconus [Fig. 6(f)] respectively. This may be due to the higher-order polynomials being very sensitive to the small, irregular surface variations of these diseased corneas. The fourth-order polynomial was hence chosen for the final surface fit in step (d) of the preprocessing procedure [see Fig. 2(d)].

#### 3.1.2 Determining the number of reference planes

As detailed in Sec. 2.2.3, we deduce axial motion associated with every A-line within B-scan images with respect to three reference image planes. To arrive at three as the optimal number of reference planes for axial motion compensation, we tested our algorithm during C-scan imaging (consisting of 512 B-scans) of an *ex vivo* bovine cornea while inducing typical axial motion of around 1 Hz (such as induced from heartbeat) and changing the number of reference planes from 2 to 4. The reference planes are distributed based on the following rule, which minimizes the compensation error (as discussed in Sec. 3.1.3): suppose there are a total of t planes and L is the length of B-scan images, the i’th plane will be located at (2i−1)L/2t. The time cost of the polynomial fitting process of the references [described by Eq. (3)] is measured and compared (using the built-in function in C++): $O(n^{3})$, in which n is the dimension of the square matrix V in Eq. (3), and it is also the number of references in our case. In our test, first-, second-, and third-order polynomials were generated to estimate axial motion based on the 2, 3, and 4 reference points (planes), respectively. The RMSE between the motion-compensated and static (reference) C-scan image (calculated as the mean value of the absolute difference between the respective A-scan corneal surface positions) was used to quantify the results.

As summarized in Table 1, three references decrease the RMSE by around 4.21  μm with only an additional 2-ms time cost, compared with two references. Although four reference planes would even further reduce the RMSE by around 0.76  μm, it requires an additional 12 ms for processing and 0.76  μm is <1  pixel and the resolution of our imaging system. Hence, the three reference-plane approaches provide an optimum compromise between performance and time cost for our overall scanning speed and considered frequency range.

**Table 1:** RMSE and time cost for C-scan imaging with different numbers of B-scan references.

Number of references	2	3	4
RMSE (μm)	6.55	2.34	1.58
Time cost (ms)	24	26	38

#### 3.1.3 Determining the reference plane positions, α

*Theory.* The compensation result also depends on the position of the reference images and the distance between the adjacent reference images, d, and thus our selection of α, which is defined as d/L, where L is the width of the raw B-scan image [see Fig. 1(b)]. Since we choose the middle reference to be fixed at the center of the x-direction, α can range from 0 (i.e., one reference plane at the center) to 0.5 (i.e., two references at the ends of the image).

**Fig. 7:**
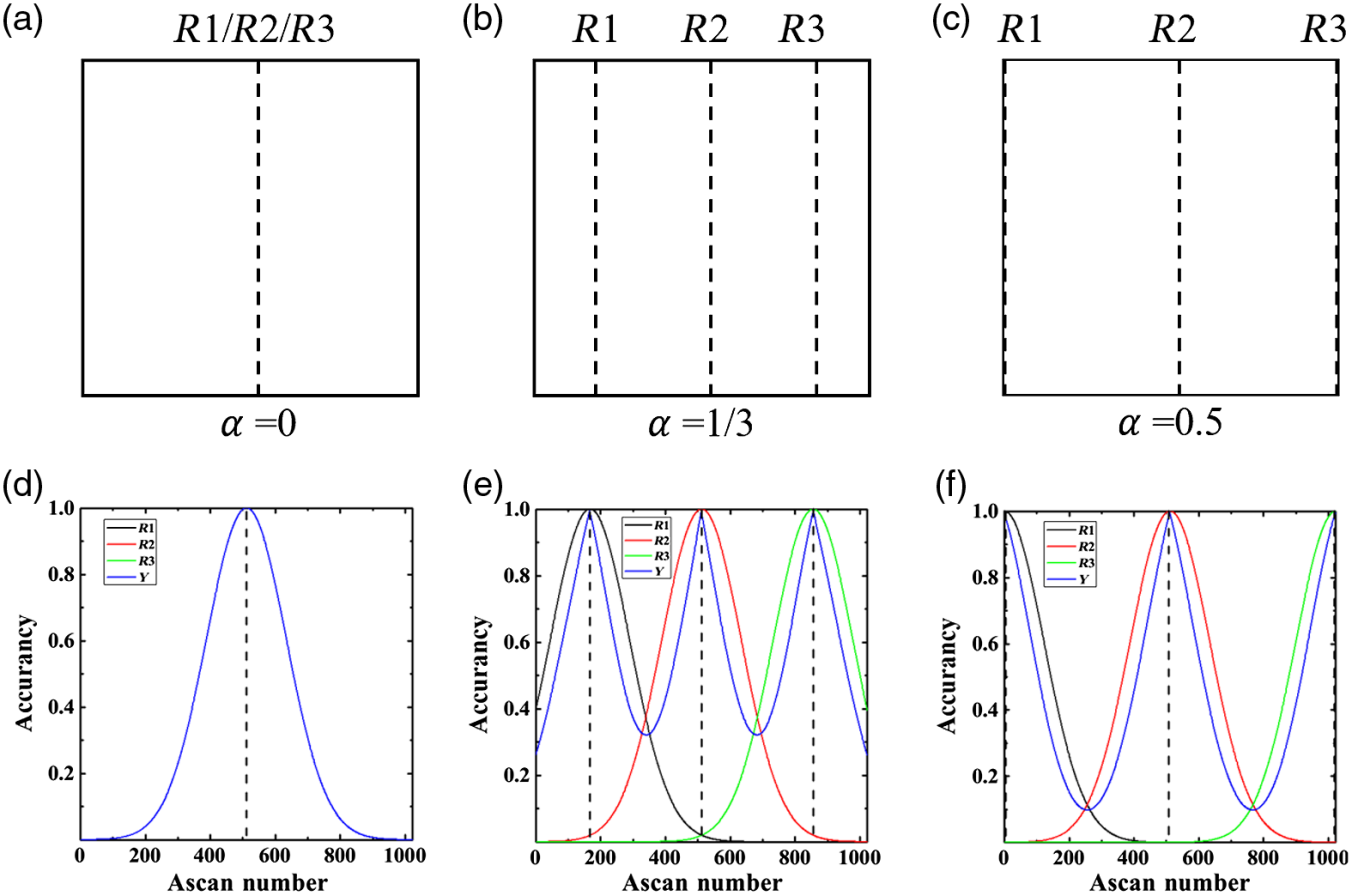
Analysis for the selection of. (a)–(c) Schematically showing the location of the three references (black dotted lines) in a B-scan image, with α set at 0, 1/3, and 0.5, respectively. (d)–(f) The corresponding accuracy distribution of A-scans within the B-scan. Black dotted lines indicate the location of the three references (R1, R2, and R3).

This is schematically shown in Figs. 7(a)–7(c), displaying the location of the three references (R1, R2, and R3; dotted lines) in a B-scan image, with α set as 0, 1/3, and 0.5, respectively.

The quantitative analysis model is based on the hypothesis that the motion compensation accuracy based on references is a Gaussian-like function of the number of A-scans within the B-scan. The fitting error within every B-scan image is considered random, so the accuracy of references used to compensate for the motion could be estimated as a Gaussian function (i.e., a widely used noise model to describe estimation-related error^26^), and our experimental result agrees with this hypothesis.

The accuracy function may be written as

$$R_{i}(x;\mu_{i},\sigma_{i})=e^{\frac{-{\left(x-\mu_{i}\right)}^{2}}{2\sigma_{i}^{2}}},$$ (8)

in which, R is the accuracy, x is the number of A-scans, ranging from 0 to 1023 in our case, and *i* is the index for three references (i=1, 2, 3). We eliminated the parameters $1/\sigma \sqrt{2\pi }$ to ensure the range of accuracy function is [0,1]. μ and σ are the mean value and the standard deviation of the Gaussian-like function, indicating the reference location and the width of the function shape, respectively. Therefore, μ of the three references can be expressed as

$$\mu_{1}=(\frac{1}{2}-\alpha )m,\;\mu_{2}=\frac{n}{2},\;\mu_{3}=(\frac{1}{2}+\alpha )m.$$ (9)

We assumed m=1023 to be the maximum number of A-scans. All three reference functions should have the same σ because all of them have the same compensation capability. Therefore, we have $\sigma =\sigma_{i}(i=1,2,3)$.

We define another accuracy function Y(x) to determine the total accuracy of the motion compensation based on more than one reference. Y(x) is a combination of several $R_{i}(x)$ and should follow two rules:

1. When α→0, $Y(x)\to R(x;\frac{n}{2},\sigma )$;
2. When α→±∞, $Y(x)\to \underset{i}{\sum }R_{i}(x;\mu ,\sigma )$.

These two rules ensure that Y(x) also ranges from [0,1]. Thus, we construct Y(x) as

$$Y(x;\mu_{i},\sigma )=\underset{i}{\sum }\frac{1/(|x-\mu_{i}|+1)}{\underset{i}{\sum }1/(|x-\mu_{i}|+1)}R_{i}(x;\mu_{i},\sigma ).$$ (10)

The number 1 in the denominator of distribution function $\left(1/(|x-\mu_{i}|)+1\right)$ is used to avoid zero. Figures 7(d)–7(f) show the accuracy functions $R_{1}(x)$, $R_{2}(x)$, $R_{3}(x)$, and Y(x) with α set to 0, 1/3 and 0.5, respectively. Among the three examples, we obtain the best accuracy result with α=1/3. The corresponding minimum accuracy of 0.33 represents the maximum error when the speed of the motion is approximately the same as the scanning rate of the reference images. However, since the reference images are obtained around 100 Hz, which is much faster than the anticipated sample motion, ranging up to 5 Hz, we expect our overall result to be highly accurate.

We also define an error function based on the accuracy function above to enable comparison with experimental data

$$E(\mu_{i},\sigma )=\overset{n}{\underset{x=0}{\sum }}[1-Y(x;\mu_{i},\sigma )].$$ (11)

Combining Eqs. (8) and (10) and assuming σ as a constant, we can write $E(\mu_{i},\sigma )$ as a function of α

$$E(\alpha ,\sigma )=\sum_{x=0}^{n}[1-Y(x;\alpha ,\sigma )].$$ (12)

**Fig. 8:**
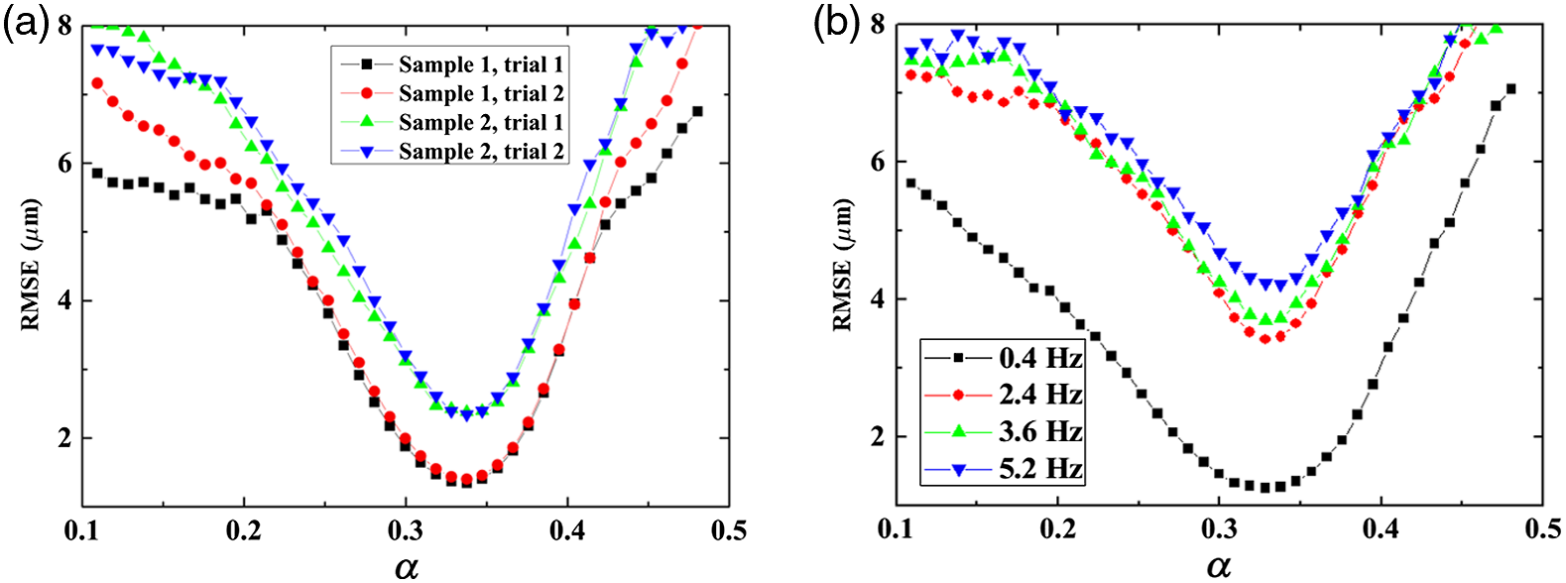
RMSE between motion-compensated and static reference images as a function of α for (a) two different *ex vivo* bovine cornea samples with two different trials with 1 Hz axial motion and (b) a phantom result with motion frequencies from 0.4 Hz to 5.2 Hz.

*Experiment.* To optimize α experimentally, we assessed the performance of the motion-compensation algorithm by performing several imaging trials while varying the distance between adjacent reference planes. The RMSE representing the difference between respective A-scan corneal surface positions of the motion-compensated and static (reference) C-scan image was used to quantify the results. Figure 8(a) shows the RMSE of the compensation result for around 1-Hz motion using two bovine corneal samples as a function of α. Two trials were performed for each sample. Figure 8(b) shows the RMSE result for different motion frequencies (0.4 to 5.2 Hz) using the corneal phantom as a function of α. Both experiments indicate that α around 0.33 achieves the best results (i.e., minimal RMSE) for all samples and frequencies used in the study. Note that, while the slower motion frequencies yielded smaller compensation errors (i.e., an RMSE of 2.34 for 1 Hz, and an RMSE of 1.26 for 0.4 Hz), even at higher frequencies (2.4 through 5.2 Hz), with α set to 0.33, our algorithm was capable of compensation with an error of <4.2  μm. Note that although the axial resolution of our system is 6  μm in air and 4.5  μm in corneal tissue, our ability to detect the peak of the point spread function is limited by the pixel size, which is 3.6  μm/pixel (for corneal tissue). Hence, 4.2  μm corresponds to an overall compensation error of about 1 pixel.

*Comparison.* To further test the performance of our model, we calculated the error function with different σ [using Eq. (10)] and compared it with the experimental data, where the difference is the fitting error defined as

$$W(\sigma )=\overset{s}{\underset{i=1}{\sum }}\frac{1}{s}|E(\alpha_{i},\sigma )-\mathrm{Expr}(\alpha_{i})|,$$ (13)

with s and Expr being the index of α and the experimental data, respectively.

**Fig. 9:**
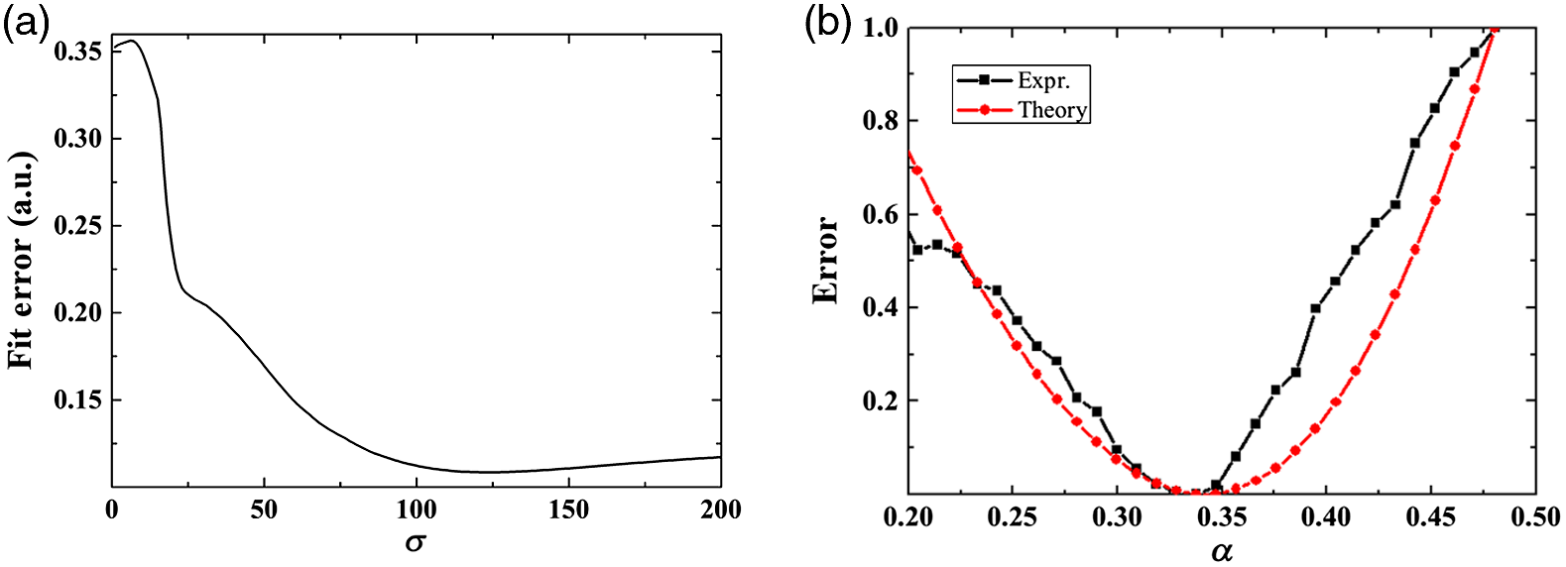
(a) Fitting error (W) between our theory and experimental data for a motion at 5.2 Hz as a function of σ. (b) Comparison between the experimental motion compensation result (black) and numerical result using the theoretical model (red) as a function of α, for minimal σ=123.

As illustrated in Fig. 9(a), the fitting error (W) achieves a minimum at σ=123. Figure 9(b) depicts the result, at minimal σ=123, as a function of α (step size 10/1024), for the experimentally obtained motion compensation result and the numerical result for a motion frequency of 5.2 Hz; note that the data has been normalized. The average absolute error difference between the theory and the experiment is 0.11. Both theory and experiment achieve a minimum error around α=0.338. Hence, we conclude that the mechanism of our motion compensation method is successfully described by our theoretical model.

The result above shows that the evenly-distributed reference planes can minimize the compensation error, which can be generalized to the distribution rule of reference planes we mentioned in Sec. 3.1.2.

### 3.2 Testing System Performance

#### 3.2.1 Fourth-order polynomial for corneal surface fit

**Fig. 10:**
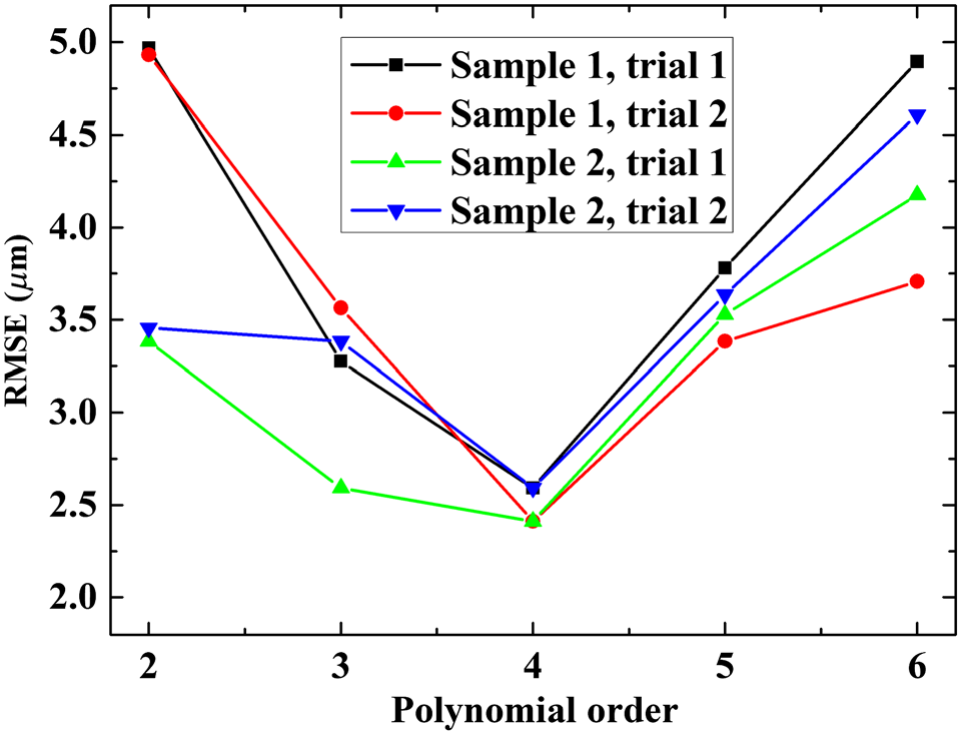
RMSE for four compensation studies using two cornea samples while inducing axial motion of 1Hz as a function of polynomial orders from 2 to 6.

To validate the selection of the fourth-order as the optimal polynomial for the corneal surface fit, we assessed the performance of the algorithm experimentally. Specifically, two imaging trials using two *ex vivo* bovine corneal samples while inducing quasisinusoidal axial motion around 1 Hz were performed. The resulting images were fitted with the different order polynomials and compared. As previously done, the RMSE was used to quantify the results. The RMSE was deduced from measuring the difference between the reference and compensated C-scan image of the sample. The results are illustrated in Fig. 10 and show that fourth-order fitting achieved the best accuracy, i.e., the minimum RMSE. Considering this and our preliminary result using clinical images of human corneas with normal and abnormal surface topology, the fourth-order polynomial was confirmed as optimal surface fit in step (d) of the preprocessing procedure [Fig. 2(d)].

#### 3.2.2 Proof-of-concept demonstration of motion compensation

Using the optimized parameters, we performed a series of volumetric imaging trials using corneal phantom (thin plastic shell) and tissue samples (*ex vivo* bovine cornea), induced with artificially generated motions, as a proof-of-concept demonstration of the proposed motion-compensation method using our in house-built 3D-OCT system.

**Fig. 11:**
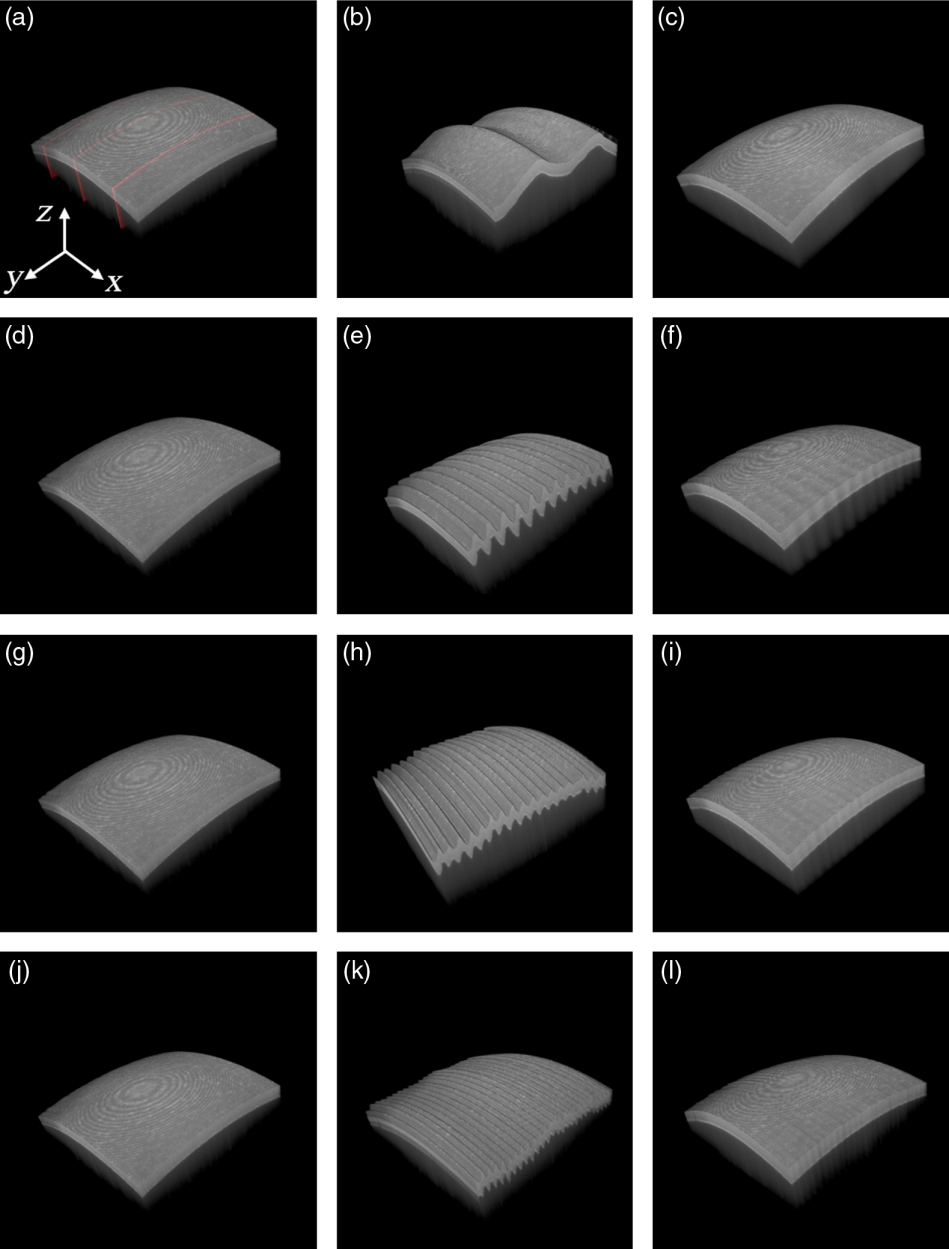
Axial motion-compensation result during volumetric (C-scan) imaging of a corneal phantom (thin plastic shell). Panels (a), (d), (g), and (j) are the static reference images of the phantom. The reference planes are indicated in red in (a). Panels (b), (e), (h), and (k) correspond to the sample motion around 0.4, 2.1, 3.6, and 5.2 Hz, respectively. Panels (c), (f) , (i), and (l) are reconstructed after using the proposed motion compensation method.

*Axial motion.* Figures 11(b), 11(e), 11(h), and 11(k) depict the volumetric (C-scan) images of the corneal phantom during induced axial motion [by means of the VTS, Fig. 1(a)] around 0.4, 2.4, 3.6, and 5.2 Hz, respectively, clearly showing the motion artifacts. Figures 11(a), 11(d), 11(g), and 11(j) are the corresponding static C-scan images, without any induced motion, that were used as references, and Figs. 11(c), 11(f), 11(i), and 11(l), depict the respective motion-compensated C-scan images using the proposed method. As expected, our algorithm successfully corrects the motion artifacts in these four motion frequency settings, with the smooth spherical surface of the phantom being visibly recovered throughout the entire volume of the sample. The RMSE, as quantitative metric of the results, is 1.3, 3.5, 3.7, and 4.2 μm for the axial motions of 0.4, 2.4, 3.6, and 5.2 Hz, respectively [see Fig. 8(b)].

**Fig. 12:**
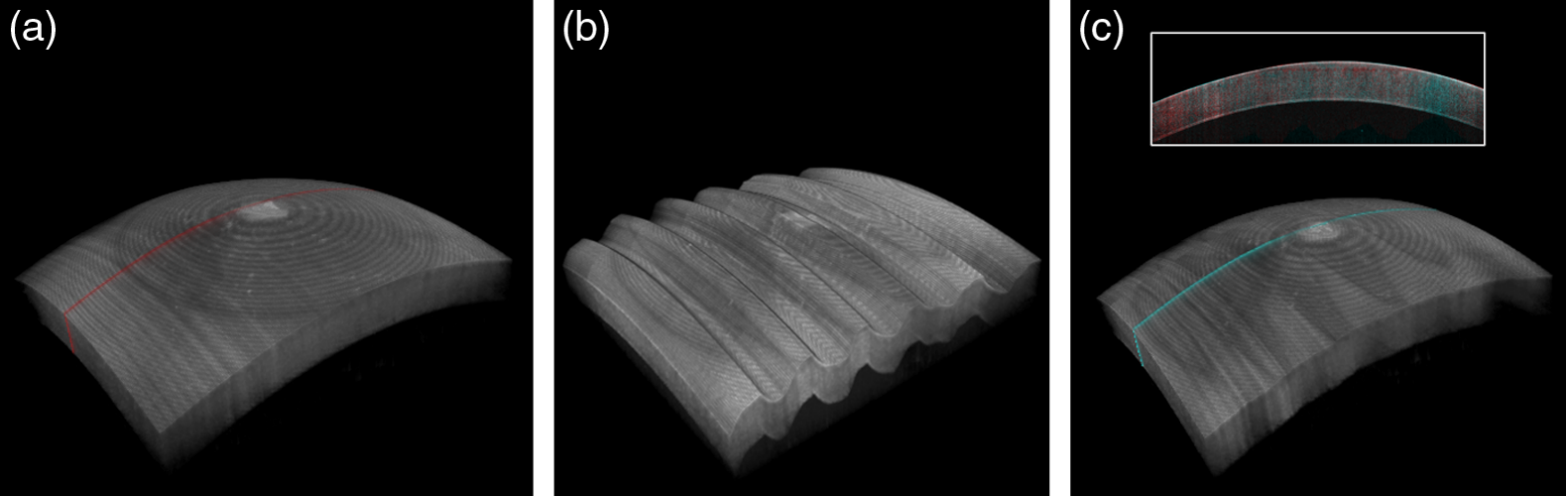
Axial motion-compensation result during volumetric (C-scan) imaging of an *ex vivo* bovine cornea. Panel (a) is the static reference of the cornea; panel (b) is the cornea with simulated axial motion of around 1 Hz. Panel (c) is the reconstructed corneal C-scan image after motion compensation. The red line in (a) and cyan line in (c) represent the corneal cross-section location of reference and compensation result, respectively. Both are also shown in the inset of (c), which provides a direct comparison between reference and postcompensation results.

An example of the axial motion-compensation result during volumetric imaging of an *ex vivo* bovine cornea is illustrated in Fig. 12. The average amplitude and frequency of the induced corneal motion were around 700  μm and 1 Hz, respectively. Figure 12(a) depicts the C-scan reference image of the cornea without any induced axial motion, whereas Figs. 12(b) and 12(c) are the C-scan images of the “moving” cornea before and after motion compensation. As expected, the motion-induced distortion of the corneal surface, clearly visible in Fig. 12(b), is successfully compensated using the proposed method [see Fig. 12(c)]. This example corresponds to trial 1 of sample 1 in Fig. 8(a), with an RMSE of 1.35  μm (for α=0.33). For direct visual comparison, the slow-axis cross-section showing the difference between reference and compensated result is also shown in the inset of Fig. 12(c).

**Fig. 13:**
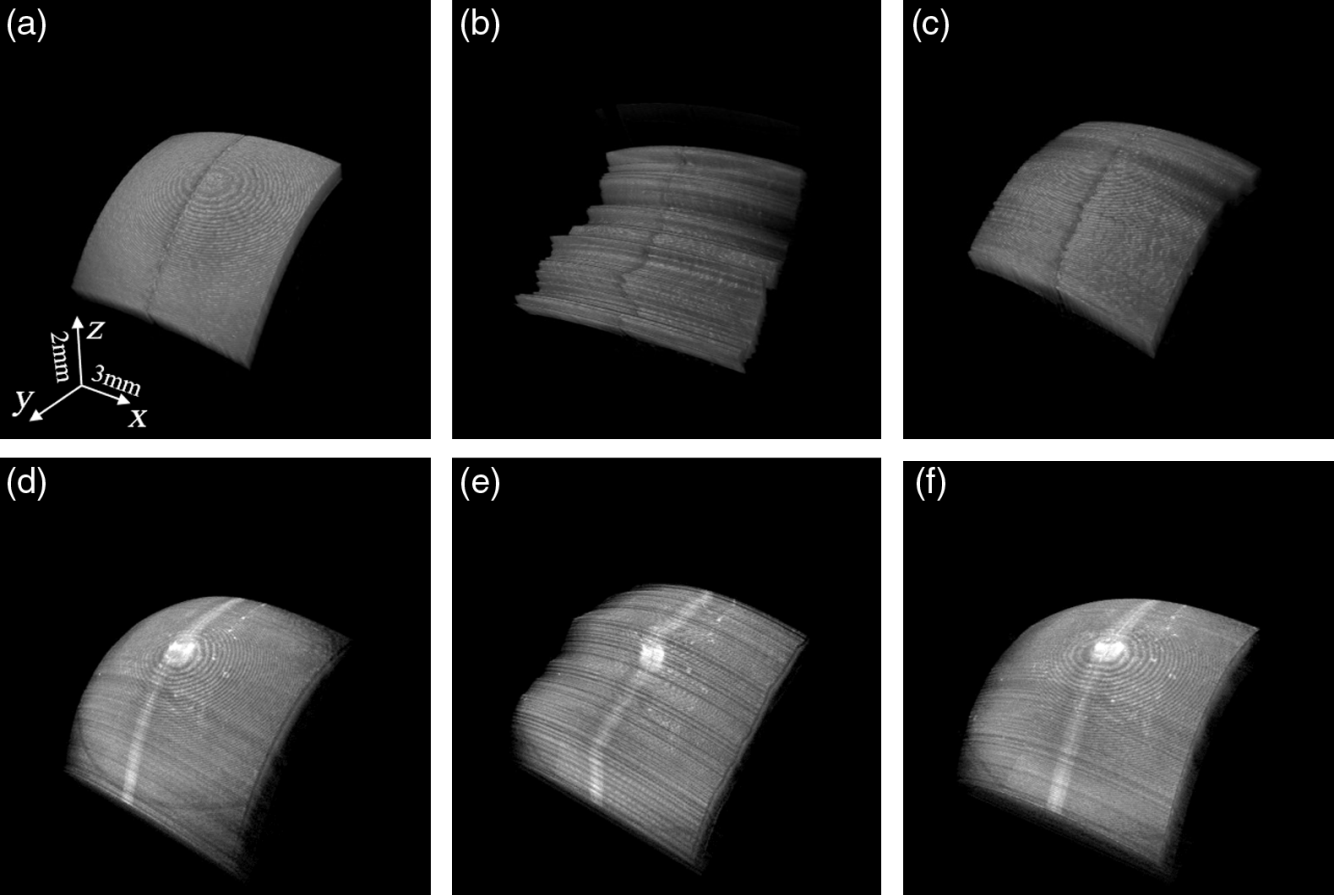
3D motion-compensation result of a phantom (top) and *ex vivo* bovine (bottom) cornea during C-scan (volumetric) free-hand imaging, i.e., with the samples positioned in the palm of a hand to simulate involuntary tissue motion in both lateral and axial directions. Panels (a) and (d) are the static reference of the cornea without any simulated motion; panels (b) and (e) are the cornea with free-hand motion. Panels (c) and (f) are the reconstructed corneal image after 3D-motion compensation.

*3D motion.* Figure 13 shows an example of the 3D motion-compensation result during C-scan (volume) “free-hand” imaging of a phantom [plastic shell; Figs. 13(a)–13(c)] and *ex vivo* bovine corneal sample [Figs. 13(d)–13(f)], i.e., with the samples positioned in the palm of the hand, to more realistically simulate the involuntary motion of living tissue occurring in both lateral and axial directions. Figure 13(a) and 13(c) depict the static (reference) C-scan image of the phantom and bovine cornea, respectively, without any motion; Figs. 13(b) and 13(e), and Figs. 13(c) and 13(f) are the C-scan images of the phantom and held corneas, before and after motion compensation, respectively. The marked central line on the corneal surface is a manually produced scar (by scratching the surface) to highlight the occurrence of lateral and axial motion during free-hand imaging [see Figs. 3(b) and 3(e)]. Note that the marked line appears straighter again after compensation [see Figs. 3(c) and 3(f)]. The RMSE of the phantom and bovine cornea result is 4.61 and 3.96  μm, respectively.

**Fig. 14:**
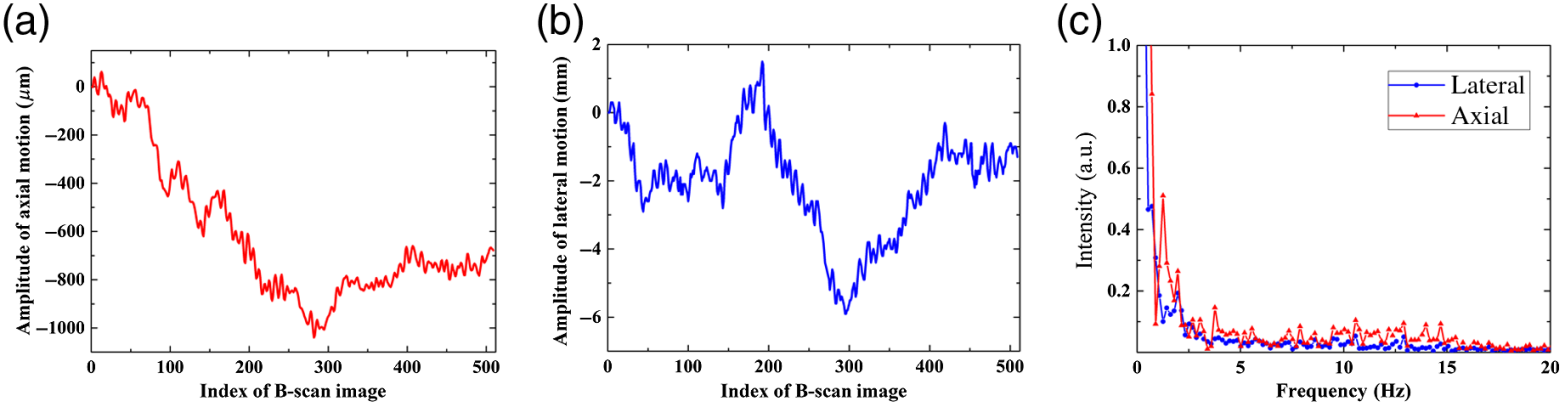
Axial and lateral motion analysis during free-hand volumetric imaging of an *ex vivo* bovine cornea. Panels (a) and (b) are the amplitude of axial and lateral tissue motion, respectively; panel (c) is the corresponding frequency information.

We also analyzed the lateral and axial motion that was induced during free-hand imaging of the corneal samples. The amplitudes of axial and lateral motion occurring during free-hand imaging of the *ex vivo* bovine cornea from Fig. 13 are depicted in Figs. 14(a) and 14(b), respectively; the corresponding frequencies are shown in Fig. 14(c). The mean value of axial and lateral motion between adjacent B-scan images is around 14.18 and 165  μm, respectively. Also note that most energy of motion, in either direction, occurs in the range from 0 to 5 Hz and that motion above 15 Hz is negligible.

### 3.3 Algorithm Efficiency

Our algorithm was first developed in MATLAB as a testbed prototype, then implemented in C++ for real-time processing. To demonstrate its efficiency and real-time capabilities, we evaluated the time consumption of the algorithm during volumetric (C-scan) imaging. The tested C-scan image contains 512 B-scans and the size of each B-scan is 1024×1024. The mean value of time consumption for each B-scan is 7.6 ms, including both preprocessing and motion compensation procedures, which is faster than our 100-Hz B-scan rate.

**Fig. 15:**
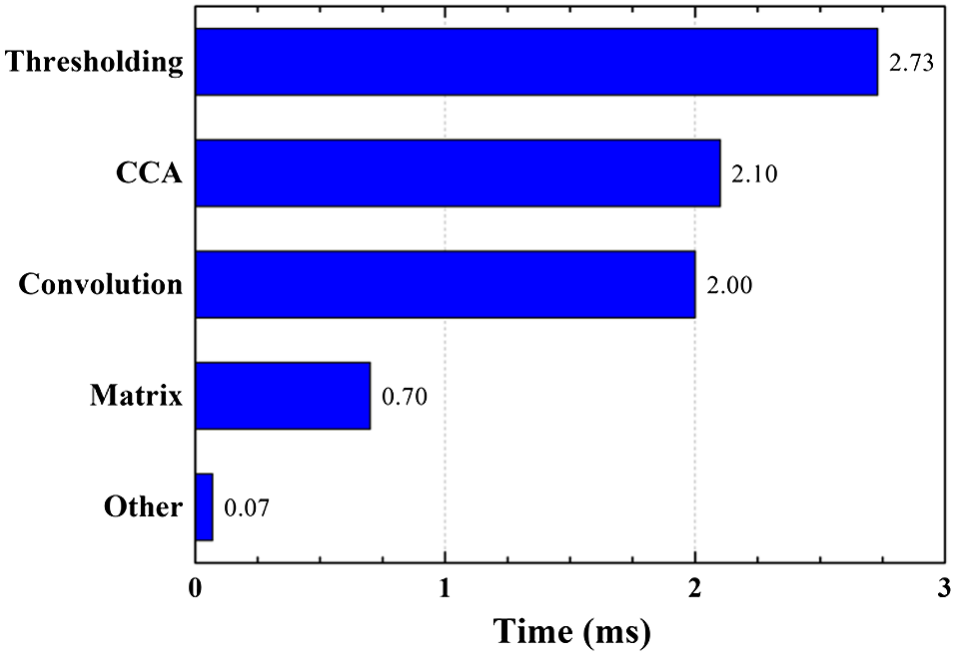
Time consumption of different processing steps in the proposed algorithm.

Detailed time consumptions for the diverse processing steps are shown in Fig. 15. Approximately 36% of the 7.6 ms are used for thresholding, 28% are used for various filter operations based on convolution, 26% are used for connect component analysis, 9% are used for matrix-related calculations, and the remaining 1% are used for other procedures. The preprocessing procedure is much more time-consuming than the actual motion compensation because the latter is based on a single variable polynomial, which only requires few computational resources.

To further improve algorithm efficiency, the GPU driven by compute unified device architecture is assigned to the thresholding task, matrix calculation, and filter operation, which are mainly responsible for the processing time. This parallel processing reduces the processing time considerably, resulting in a mean value of overall time consumption for each B-scan image preprocessing and motion compensation of 3.40 ms, including data I/O between CPU and GPU.

A single B-scan image is processed simultaneously during the acquisition of the subsequent B-scan frame. With the processing time (3.40 ms) being much smaller than the actual acquisition time (10 ms, set by the OCT engine), every motion-compensated B-mode frame is processed and displayed in real-time, at the same speed as the image acquisition.

## 4 Discussion

We implemented an OCT system for volumetric imaging of the cornea, capable of compensating both axial and lateral motion using a combination of intensity and topological analysis, with the corneal surface being identified and fitted to a polynomial curve and compared with three orthogonal references (slow-axis) B-scans initially obtained to deduce the magnitude of motion-related shifts to correct the fast-axis B-cans throughout the OCT volume.

The use of orthogonal B-scans to compensate for motion in OCT volumes is not new, especially for the retina. For example, Kraus et al.^13^ retrospectively gathered additional B-scans along the slow axis of the acquired OCT volume to align the fast-axis (orthogonal) B-scans, on a per A-scan basis. Other related retinal methods include those by Lezama et al.,^27^ who also used a segmentation approach, and by Kim et al.^18^ who performed simultaneous orthogonal scanning by means of two perpendicularly polarized beams. Our method could also be applicable for retinal imaging, including OCT angiography (OCT-A), upon redesigning the scanning and microscope objective and imaging system of the OCT apparatus. Note that due to the refractive power of the cornea and crystalline lens, the OCT image of the retina is distorted and does not represent the true curvature of the retina so that additional image processing is required to obtain the true reference image. However, for many applications (including robotic tool guidance that we are pursuing^2^^,^^28^^–^^30^), an optically distorted image is sufficient and only the relative position and the curvature are needed to correctly assess the tool to tissue distance. Therefore, even for retinal imaging, one could still use the same processing methods to obtain motion-free retinal images.

An advantage of our approach is that every target surface is represented as a polynomial curve, so the difference between different B-scans can be calculated with low computational cost by the comparison of polynomial parameters rather than complicated and time-consuming cross-correlation,^18^^,^^27^ or local pairwise phase correlation.^31^ Although the simulated motion in this preliminary (*ex-vivo*) proof-of-concept study does not reflect the complex movements of the living eye, most involuntary and physiological ocular (lateral and axial) motion is expected to fall within the range from 0 to 5 Hz (including micro saccades^32^), which is much smaller than our B-scan rate of 100 Hz. Hence, sample movement within each B-scan image could be approximated by a second-order polynomial curve, only requiring three references and reducing scanning time cost.

The use of higher-order polynomial, rather than spherical approximations,^5^ for corneal surface fits, also ensures the recognition and differentiation of involuntary tissue movement from abnormal surface topology, which may be particularly important for imaging in patients with corneal disease.

Similarly, with one of the goals of developing the algorithm has been to use the motion-compensated imagine system for ophthalmic applications in patients (e.g., to guide the robotics eye surgery and/or to extract quantitative information from volumetric measurements), a raster scan was favored over a radial-scan approach for its ease of use (e.g., to locate and track the movement of the surgical tool, such as needles).

Limitations of the current approach include the assumption of no motion occurring during the acquisition of the three reference (slow-axis) B-scans. In fact, the observed increase in RMSE with higher-frequency motion [Fig. 8(b)] could be the result of higher-velocity movements affecting the references and may become enhanced during *in-vivo* application of the approach, in particular during imaging of patients whose involuntary eye motion may be exaggerated.^10^ To reduce potential motion artifacts during reference acquisition, we plan to decrease the number of A-scans during the acquisition of the references (slow-axis B-scan images) during future *in vivo* studies. For example, the current size of a reference B-scan is 512×1024, with an acquisition time of 5 ms, which is shorter than the typical (fast-axis) B-scan image acquisition time of 10 ms. However, further reducing the reference B-scans to only 100 A-scans would still be sufficient to perform the motion compensation using our algorithm and reducing the acquisition time for each reference to around 1 ms so that motion during reference-plane acquisition could indeed be considered negligible.

The algorithm could also be accelerated by choosing a smaller region of interest (ROI). Our C-scan data composed of 1024×512×1024  voxels (X×Y×Z) corresponds to a 1  cm×1.1  cm×3.7-mm corneal tissue volume. If we reduced the ROI to around 0.5  cm×0.65  cm×3.7  mm with the same image resolution, e.g., we would only spend one-fourth time for the image acquisition and processing and 1/2 time for reference acquisition. This would also reduce the effect of motion, in particular higher-frequency motion that might be pronounced during patient (*in-vivo*) imaging, and lead to higher compensation accuracy.

Future work will include such optimizations to address these limitations, enhance the approach and optical system to be more clinically oriented (including accounting for more complex motion of the living eye), and test system performance during *in vivo* measurements.

## 5 Conclusion

We described a 3D motion-compensated OCT system based on higher-order regression toward real-time volumetric imaging of the cornea. Fourth-order polynomial surface fitting in combination with the acquisition of three orthogonal (slow-axis) reference planes ensures differentiation of abnormal surface topology from involuntary tissue motion. Volumetric (C-scan) imaging of corneal phantom and bovine samples during simulated (axial and free-hand) motion demonstrated effective compensation of 3D motion artifacts with an overall error of <4.61  μm. The processing time for each (fast-axis) B-scan is 3.40 ms using a GPU-based parallel computation technique. Our approach can be expanded to 3D-imaging of other ocular tissues. Implementing such motion-compensation strategies into clinical OCT systems has the potential to improve the reliability of objective and quantitative information that can be extracted from volumetric OCT measurements.
